# Inhibition of Immune Checkpoints and Vascular Endothelial Growth Factor as Combination Therapy for Metastatic Melanoma: An Overview of Rationale, Preclinical Evidence, and Initial Clinical Data

**DOI:** 10.3389/fonc.2015.00202

**Published:** 2015-09-22

**Authors:** Patrick A. Ott, F. Stephen Hodi, Elizabeth I. Buchbinder

**Affiliations:** ^1^Department of Medical Oncology, Melanoma Disease Center, Center for Immuno-Oncology, Dana-Farber Cancer Institute, Harvard Medical School, Boston, MA, USA; ^2^Department of Medicine, Brigham and Women’s Hospital, Harvard Medical School, Boston, MA, USA

**Keywords:** PD-1:PD-1L blockade, angiogenesis inhibitors, VEGF, melanoma, immunotherapy

## Abstract

The role of angiogenesis as a mediator of immune regulation in the tumor microenvironment has recently come into focus. Furthermore, emerging evidence indicates that immunotherapy can lead to immune-mediated vasculopathy in the tumor, suggesting that the tumor vasculature may be an important interface between the tumor-directed immune response and the cancer itself. The advent of immune checkpoint inhibition as an effective immunotherapeutic strategy for many cancers has led to a better understanding of this interface. While the inhibition of angiogenesis through targeting of vascular endothelial growth factor (VEGF) has been used successfully for the treatment of cancer for many years, the mechanisms of its anti-tumor activity remain poorly understood. Initial studies of the complex relationship between angiogenesis, VEGF signaling and the immune system suggest that the combination of immune checkpoint blockade with angiogenesis inhibition has potential. While the majority of this work has been performed in metastatic melanoma, immunotherapy is rapidly showing promise in a broad range of malignancies and efforts to enhance immunotherapy will broadly impact the future of oncology. Here, we review the preclinical rationale and clinical investigations of combined angiogenesis inhibition and immunotherapy/immune checkpoint inhibition as a potentially promising combinatorial approach for cancer treatment.

## Introduction

Immune checkpoint blockade with monoclonal antibodies directed against CTLA-4, PD-1, and PD-L1 has shown striking anti-tumor activity in an increasing number of solid tumors and hematologic malignancies, including tumors previously not considered immune responsive. However, many patients with advanced cancer still do not receive clinical benefit from these treatments. The objective tumor response rates seen in patients with advanced melanoma treated with concurrent ipilimumab and nivolumab suggest that significant improvements are achievable with combinatorial approaches (1–3); however, the combinatorial regimen is associated with significant immune-related toxicities. Alternative methods to improve the anti-tumor activity of immune checkpoint blockade without substantially increasing toxicity are, therefore, desirable.

The PD-1/PD-L1 pathway appears to be critical in downregulating presumably *in vivo* primed tumor-directed T cell responses as demonstrated by the successes of PD-1/PD-L1 inhibition. Nevertheless, additional immunosuppressive mechanisms within the tumor microenvironment that compromise melanoma-directed T cell responses have been identified and validated. These include infiltration with inhibitory cells, such as T regulatory cells (Tregs) (4, 5), myeloid-derived suppressor cells (MDSCs), and tumor-associated macrophages; upregulation of additional inhibitory receptors, such as TIM-3 (6) and LAG-3 (7); and the secretion of immunosuppressive soluble factors, such as cytokines and chemokines. Unfortunately, while the immunosuppressive mechanisms of immune checkpoint blockade are being uncovered, good predictive biomarkers of success have yet to be validated (8, 9). These inhibitory mechanisms likely limit the efficacy of many immunotherapies, and provide targets for combination therapy.

The vascular endothelial growth factor (VEGF) has been recognized as a critical mediator of immune suppression, suggesting that VEGF blockade, which has proven effective for the treatment of several cancers, may have a favorable impact on the anti-tumor immune response in addition to its direct effects on the tumor vasculature. Moreover, our own studies in tumor samples obtained from advanced melanoma patients after treatment with ipilimumab revealed immune-mediated vasculopathy associated with tumor necrosis and heavy infiltration with mononuclear cells (10). These findings indicate that CTLA-4 inhibition may directly modulate tumor vessels in addition to its effect on the activation of T cells (10). We have also recently shown that high serum levels of VEGF (sVEGF) are associated with decreased overall survival in advanced melanoma patients treated with ipilimumab, suggesting that sVEGF levels may predict outcomes after immune checkpoint inhibition (11).

By facilitating both the growth of cancer cells and immune suppression, tumor angiogenesis is an important link between a tumor and the immune response directed at that tumor. Angiogenic factors have been shown to regulate trafficking across tumor endothelia (12). Consequently, targeting angiogenesis may be an effective strategy to increase the efficacy of primarily T cell-directed immunotherapy, such as immune checkpoint blockade. Preclinical evidence of this important interplay between tumor, tumor vasculature, and immune cells as well as recently emerging clinical data is reviewed.

## Rationale for Targeting the Vascular Endothelial Growth Factor Pathway in Combination with T Cell-Directed Immunotherapy in Melanoma

Vascular endothelial growth factors are involved in angiogenesis, lymphangiogenesis, and vasculogenesis and are primarily known as mediators of tumor neovascularization (13, 14). Different isoforms of VEGF (A–F) bind to transmembrane receptors (VEGF-R 1–3), resulting in dimerization and activation through phosphorylation of tyrosine kinases. VEGF is secreted by most tumors in response to hypoxia-inducible factor (HIF) or upregulation of oncogenes, such as c-myc (15–17). Increased VEGF serum levels are associated with a poor prognosis in patients with malignancy (18). Melanoma cells demonstrate high levels of expression of VEGF, VEGF-R1, VEGF-R2, and VEGF-R3 and high circulating serum levels of VEGF are associated with poor prognosis in patients with melanoma (19, 20). The anti-VEGF antibody bevacizumab given in combination with chemotherapy (carboplatin/paclitaxel) has demonstrated a promising activity in advanced melanoma patients treated in a phase 2 trial (21). Aflibercept (VEGF Trap), a fusion protein combining the Fc portion of human IgG with the extracellular ligand-binding domains of human VEGFR1 and VEGFR2, which acts as a decoy VEGF receptor has shown promising single agent activity in early studies in melanoma (22, 23). Furthermore, high levels of soluble VEGF have been associated with a lack of response to high-dose IL-2 therapy and decreased OS in cancer patients treated with ipilimumab (11, 24).

**Figure 1:**
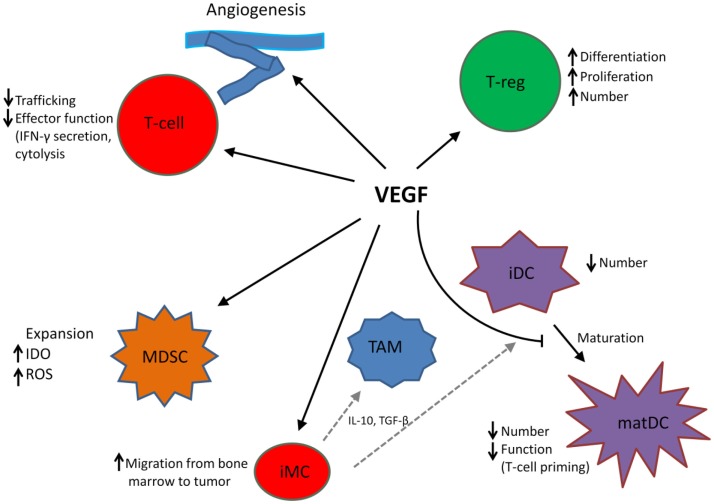
**VEGF modulates the function of T cells, suppressive immune cells, and stroma in the tumor microenvironment, leading to an immunosuppressive state**. MDSC, myeloid-derived suppressor cell; iDC, immature dendritic cell; matDC, mature dendritic cell; TAM, tumor-associated macrophage; T-reg, T-regulatory cell; iMC, immature myeloid cell; TAM, tumor-associated macrophage. Dotted gray lines indicate differentiation from iMC to TAM and iDC, respectively.

In addition to its role in angiogenesis, VEGF modulates anti-tumor immunity on multiple levels including promotion and expansion of inhibitory immune cell subsets, such as Tregs and MDSCs, suppression of dendritic cell (DC) maturation, mitigation of effector T cell responses, and alteration of lymphocyte development and trafficking (25). See Figure 1.

## VEGF Promotes Suppressive Immune Cell Populations

### T regulatory cells

T regulatory cells inhibit effective anti-tumor responses and are generally associated with poor outcomes in cancer patients (26). Although VEGF-A can lead to Treg differentiation by generating immature DCs (27), VEGF was also shown to directly induce Treg proliferation in the CT26 colorectal cancer model (28). In tumor-bearing mice, the percentage of Tregs among CD4^+^ T cells was significantly enhanced in the spleen, VEGF inhibition resulted in decrease of Treg cells to normal numbers. Furthermore, in patients with metastatic colorectal cancer, peripheral levels of VEGF-A were elevated and the percentage of Tregs were higher than in healthy volunteers and decreased in response to bevacizumab treatment (28). Conversely, Tregs can directly promote tumor angiogenesis. In a mouse ovarian cancer model, tumor hypoxia resulted in the recruitment of Tregs through upregulation of the chemokine ligand 28 (CCL28), leading to increased tumor growth (29, 30).

### Myeloid-derived suppressor cell

Myeloid-derived suppressor cells represent a heterogeneous population of immature myeloid cells that have been prevented from fully differentiating into mature cells during pathological conditions, such as cancer and inflammation. MDSC inhibit tumor-directed T cell responses through a variety of mechanisms including depletion of arginine and production of NO, indoleamine 2,3-dioxygenase, and reactive oxygen species (ROS). VEGF has been shown to promote the expansion of “MDSCs” (31) and decreased numbers of CD11b^+^ VEGFR1 + MDSCs in the peripheral blood were observed in the peripheral blood of renal cell cancer (RCC) patients treated with the anti-VEGF antibody bevacizumab. In a mouse model, Gr1^+^CD11b^+^ immature myeloid cells accumulated in the spleen, whereas CD4 and CD8 cells decreased in mice treated with VEGF; this effect could be completely reversed by blocking VEGFR-2 (32).

## VEGF Leads to Compromised Antigen-Presenting Cell and T Effector Cell Function

### Dendritic cells and tumor-associated macrophages

Antigen-specific cells are critical for the induction of effective T cell responses and inadequate presentation of tumor antigens by host antigen-presenting cells is one mechanism of immune escape. VEGF was found to limit the maturation of DC precursors into mature DC capable of presenting tumor antigens and inducing a T cell response directed at tumor antigens (31, 33). VEGFR1 appears to be the primary mediator of the VEGF inhibition of DC maturation, while VEGFR2 plays a role in early hematopoietic differentiation, but is less important in the final stages of DC maturation (34). Signaling through VEGFR1/Flt-1 inhibits the activation of NF-kappa B in hematopoietic progenitor cells, which has been implicated in the generation of mature DCs (35). The expression of VEGF negatively correlates with DC numbers in tumor tissue and peripheral blood of patients with different types of cancer.

Vascular endothelial growth factor was also found to attract immature myeloid cells from the bone marrow into tumor sites. Once in the tumor, these precursors develop into immature DC or tumor-associated macrophages under the influence of VEGF and other immunosuppressive soluble factors, such as IL-10 and TGF-β. Tumor-associated macrophages can also be directly recruited to the tumor by VEGF. Increased numbers of TAM and elevated levels of VEGF were associated with metastasis in a melanoma model (36).

Inhibition of VEGF with bevacizumab leads to a decrease in immature progenitor cells and a modest increase in the DC population in the peripheral blood of cancer patients (37). In animal tumor models, VEGF therapy increased the number and function of DC detected in lymph nodes and spleens. Treatment of tumor-bearing mice with an anti-VEGF antibody at a dose that did not block tumor growth directly, but suppressed serum VEGF levels, increased mature DC numbers, improved DC function, and resulted in a pronounced decrease in tumor growth that was associated with an enhanced tumor-specific CTL response (38).

### T effector cells

Studies in animal models suggest that VEFG interferes with T cell development from hematopoietic progenitor cells, thus further impairing immune responses (39). Exposure to VEGF at levels observed in advanced cancer led to decrease CD4^+^/CD8^+^ thymocytes and lymphoid progenitors (39). In a mouse model, CD4 and CD8 cell numbers decreased in the spleen of mice treated with VEGF, while Gr1^+^CD11b^+^ immature myeloid cell numbers accumulated; this effect could be reversed by blocking VEGFR-2. In addition, VEGF-mediated inhibition of T cell development in the thymus was also reversed by VEGFR-2 inhibition (40).

## VEGF Effects on Tumor Vasculature and Lymphocyte Trafficking

Tumor vasculature is often markedly abnormal leading to a hypoxic and acidic microenvironment with high interstitial fluid pressure (41). Effective anti-cancer immune responses depend upon the ability of tumor-reactive T cells to infiltrate into a tumor (42–44). One barrier to effective tumor T cell infiltration is the endothelium of the tumor vasculature, limiting adhesive interactions between endothelial cells and T cells (45, 46). Angiogenic factors suppress expression of endothelial intercellular adhesion molecule-1 causing a reduction in leukocyte adhesion to endothelial cells (47). VEGF blockade restores these endothelial-T cell interactions through effects on vascular cell adhesion molecule 1 (VCAM-1) and intracellular adhesion molecule 1 (12, 48). Antiangiogenic treatment can lead to a normalization of tumor vasculature with regards to anatomy and function. This regeneration of an intact vasculature can have beneficial effects on trafficking of tumor-specific T cells and other immune effectors (49, 50).

Fas ligand (FasL) is a mediator of T cell apoptosis; in the majority of tumors tested in a tissue array including a wide spectrum of tumor types, FasL was found to be selectively expressed on tumor endothelium, but not on the tumor cells themselves. Moreover, in human ovarian cancer tissue samples, FasL expression was significantly higher on endothelial cells within tumor islets as compared to endothelial cells in stroma, providing an explanation for an earlier observation of T cell infiltrates preferentially seen in tumor stroma. In human tumors, FasL expression has been linked to absence of intratumoral CD8^+^ T cells, but not Tregs, and endothelial cells expressing FasL were shown to kill T effector cells. Furthermore, VEGF-A, IL-10, and prostaglandin E_2_ secreted by microvascular endothelial cells (HMVECs) *in vitro* induced endothelial cell FasL expression. In mouse models, FasL lead to the preferential killing of tumor-reactive CD8^+^ T effector cells, but not Treg cells, because of higher anti-apoptotic gene expression on Treg cells. Blocking FasL or VEGF with antibodies resulted in marked increase in tumor infiltration with CD8^+^ cells (48).

## Synergy of VEGF Inhibition and Immunotherapy: Preclinical Models

In an adoptive T cell transfer (ACT) model, mice bearing large established B16 tumors were treated with pmel-1 T-cell receptor (TCR) transgenic T cells, which specifically recognize the melanocyte differentiation antigen gp100. When ACT was combined with an anti-VEGF-antibody, there was significantly increased anti-tumor activity over ACT alone, whereas anti-VEGF therapy, by itself, had no treatment effect. Experiments using luciferase-expressing pmel T cells showed that a single dose of anti-VEGF antibody given 2 days prior to ACT resulted in significantly enhanced infiltration of pmel T cells into tumors, suggesting that the augmented anti-tumor activity was mediated by more effective trafficking of T cells into the tumors (51). In a separate study, mice bearing B16 melanoma were treated with a GM-CSF-secreting tumor cell vaccine in combination with VEGF blockade using a chimeric adeno-associated virus vector expressing soluble VEGF receptor (sVEGFR1/R2). Tumor vaccine in combination with sVEGFR1/R2 leads to prolonged survival over tumor vaccine alone. Furthermore, tumors of mice treated with the combination had significantly increased frequencies of activated DCs and effector T cells, whereas Treg numbers were decreased (52).

## CTLA-4 Inhibition Combined with VEGF-Blockade: Clinical Experience in Advanced Melanoma

A phase I study using ipilimumab plus bevacizumab conducted at our institution provides the first analysis of immune checkpoint blockade and angiogenesis inhibition in cancer patients (53). Forty-six patients with advanced melanoma received ipilimumab (at either 3 mg or 10 mg/kg) every 3 weeks for four doses (induction phase) and bevacizumab (7.5 mg/kg or 15 mg/kg) every 3 weeks. After the induction phase, ipilimumab was given every 12 weeks (maintenance phase) and bevacizumab was continued every 3 weeks. The primary endpoints were safety and preliminary efficacy. Grade 3 and 4 toxicities were observed in 11 patients (23.9%) and included colitis, hepatitis, uveitis, and giant cell arteritis. Eight patients had partial responses and 22 patients had stable disease (disease control rate 30/46, 67%).

On treatment, tumor biopsies showed intense infiltration with CD8^+^ T cells and CD163^+^ dendritic macrophages within the tumor vasculature. Patients treated with ipilimumab alone who had biopsies performed had substantially less infiltration with CD8^+^ T cells and CD163^+^ macrophages. Morphologic changes of the tumor vasculature with columnar and rounded CD31^+^ cells were observed post-treatment with ipilimumab plus bevacizumab. Increased expression of E-selectin was seen with combined treatment compared to ipilimumab monotherapy, indicating endothelial activation. The types of changes seen were similar to alterations in high endothelial venules seen in secondary lymphoid organs and associated with lymphocyte extravasation, suggesting an improved ability of lymphocytes to migrate into tumor tissues. Furthermore, treatment with ipilimumab plus bevacizumab leads to increased numbers of circulating memory CD4 and CD8 cells (CCR7^+/−^CD45RO^+^) in the peripheral blood compared to ipilimumab alone.

Galectins are a family of carbohydrate-binding proteins with an affinity for β-galactosides. Galectin-1 was found to play a role in immune regulation. Galectin-1 binding to Gal-1 ligands on immune and endothelial cells associated with melanoma causes dampened immune response and increased angiogenesis in melanoma (54). Increased antibody levels directed to the galectins 1, 3, and 9 found in post-treatment sera indicate that one mechanism by which combined CTLA-4 and VEGF blockade may increase immune regulation is through inhibition of galectin. This inhibition has potential implications for both immune regulation and angiogenesis.

These early promising clinical results with correlative work confirming mechanisms of synergy between immune checkpoint inhibition and antiangiogenic therapies have led to further testing. There are several trials combining immunotherapy and bevacizumab in melanoma including a randomized phase II trial with ipilimumab (ECOG trial E3612, NCT01950390), several trials with anti-PD1 antibodies (NCT02210117 and NCT02348008), and a phase Ib with anti-PDL1 antibody (NCT01633970). In addition, a phase I trial of the anti-PD1 antibody pembrolizumab plus Ziv-Aflibercept in patients with Advanced Solid Tumors is ongoing (NCT02298959). Receptor tyrosine kinase inhibitors with numerous targets, which include VEGF, have activity in RCC, hepatocellular carcinoma, gastrointestinal stromal tumors, and other malignancies. There are numerous trials ongoing combining these agents with immune checkpoint blockade. Other angiogenic targets beyond VEGF are being explored in early clinical testing. Angiopoietin 2 (Ang2) is a growth factor expressed in a variety of tumor types, which correlates with increased angiogenesis and poor prognosis (55). A trial combining MEDI3617 and anti Ang2 antibody and tremelimumab (anti-CTLA4) in melanoma is enrolling participants (NCT02141542). See Table 1 for a listing of active trials.

**Table 1:** **Clinical trials combining immune checkpoint blockade and angiogenesis inhibition**.

Checkpoint inhibitor	Angiogenesis inhibitor	Tumor type	Design	Status	ID no.
Ipilimumab	Bevacizumab	Melanoma	Phase 1, multiple cohorts	Completed	NCT00790010
Ipilimumab	Bevacizumab	Melanoma	Phase 2, randomized	Recruiting	NCT01950390
MPDL-3280A	Bevacizumab	Solid tumors	Phase 1, multiple cohorts	Recruiting	NCT01633970
Nivolumab	Bevacizumab	RCC	Phase 2, randomized	Recruiting	NCT02210117
Nivolumab	Bevacizumab	NSCLC	Phase 1, randomized, multiple cohorts	Recruiting	NCT01454102
Pembrolizumab	Bevacizumab	RCC	Phase 1b/2	Recruiting	NCT02348008
Pembrolizumab	Bevacizumab	NSCLC	Phase 1 and 2, multiple cohorts	Recruiting	NCT02039674
Pembrolizumab	Bevacizumab	High Grade Glioma	Phase 1 (+HFSRT)	Recruiting	NCT02313272
Pembrolizumab	Bevacizumab	GBM	Phase 2	Recruiting	NCT02337491
Pembrolizumab	Ziv-Aflibercept	Solid tumors	Phase 1	Recruiting	NCT02298959
Tremelimumab	MEDI3617 (anti-ang-2)	Melanoma	Phase 1	Recruiting	NCT02141542

*NSCLC, non-small cell lung cancer; RCC, renal cell cancer; GBM, glioblastoma multiforme; HFSRT, hypofractionated stereotactic irradiation; ang-2, angiopoietin 2*.

## Conclusion

The remarkable successes of immune checkpoint inhibition in an increasing number of tumors have brought immunotherapy to the forefront of cancer treatment in recent years. Nevertheless, while overall response rates of 50% and higher, with many responses being durable, have been seen in some tumor types, such as melanoma and Hodgkin’s lymphoma, the efficacy is much lower or absent in other tumor types. Novel and combinatorial approaches are, therefore, necessary to improve outcomes with checkpoint inhibition. VEGF plays a central role in suppressing tumor-directed immune responses and promoting angiogenesis. Modulating this suppressive state in the tumor microenvironment through angiogenesis inhibition is an attractive partnering strategy for immune checkpoint inhibitors. Combined therapy with ipilimumab and bevacizumab in melanoma patients resulted in encouraging anti-tumor activity and had beneficial effects on the host anti-tumor immune response, including increase of memory cells in the peripheral blood, increased effector cell trafficking, and enhanced antibody responses to galectins. Many trials are underway exploring the concept of checkpoint inhibition in combination with angiogenesis inhibition, including VEGF blockade and inhibition of novel angiogenesis targets, such as angiopoietin 2.

## Conflict of Interest Statement

The authors declare that the research was conducted in the absence of any commercial or financial relationships that could be construed as a potential conflict of interest.
